# Carbon-11 carboxylation of trialkoxysilane and trimethylsilane derivatives using [^11^C]CO_2_†
†Electronic supplementary information (ESI) available. See DOI: 10.1039/d0cc00449a


**DOI:** 10.1039/d0cc00449a

**Published:** 2020-03-25

**Authors:** Salvatore Bongarzone, Nicola Raucci, Igor Camargo Fontana, Federico Luzi, Antony D. Gee

**Affiliations:** a School of Biomedical Engineering & Imaging Sciences , King's College London , King's Health Partners , St Thomas’ Hospital , London SE1 7EH , UK . Email: salvatore.bongarzone@kcl.ac.uk ; Email: antony.gee@kcl.ac.uk

## Abstract

A novel carboxylation radiosynthesis methodology is described starting from cyclotron-produced [^11^C]CO_2_ and fluoride-activated silane derivatives. Six carbon-11 labelled carboxylic acids were obtained from their corresponding trimethylsilyl and trialkoxysilyl precursors in a one-pot labelling methodology. The radiochemical yields ranged from 19% to 93% within 12 minutes post [^11^C]CO_2_ delivery with a trapping efficiency of 21–89%.

## Abstract 2

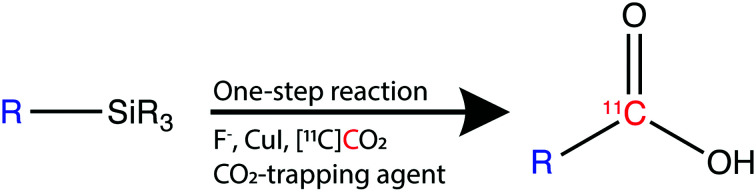
A simple and rapid carbon-11 carboxylation radiosynthesis method.

## 

Carbon-11 (^11^C) is a short-lived radionuclide (*t*_1/2_ = 20.4 min) commonly applied in positron emission tomography (PET) imaging.^1^ The isotopic substitution of carbon-12 for a carbon-11 atom in bioactive molecules maintains the chemical and biological properties of the non-radioactive autologue, allowing the study of the pharmacokinetics and biodistribution of a wide range of biologically active molecules in living subjects.^1^

^11^C is cyclotron-produced in the form of carbon dioxide ([^11^C]CO_2_) which can be directly incorporated into various biologically relevant molecules, such as [*carbonyl*-^11^C]carboxylic acids.^2^ Traditionally, aromatic carbon-11 labelled carboxylic acids have been labelled directly from [^11^C]CO_2_ using either (i) Grignard reagents^3^ or (ii) aromatic boronic esters as supporting reagents (Scheme 1).^4^

**Scheme 1:**
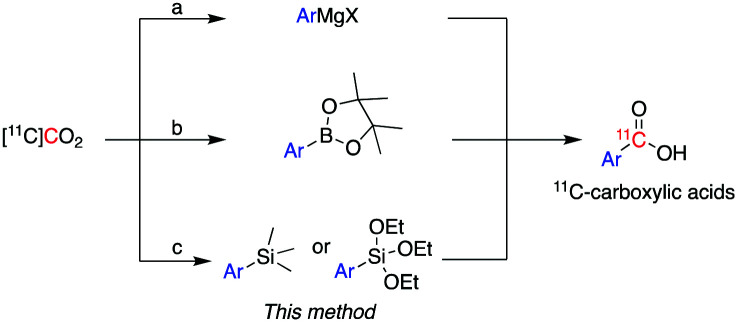
Current methods for the preparation of aromatic carbon-11 labelled carboxylic acids from [^11^C]CO_2_ using: (a) Grignard reagents, (b) boronic esters and (c) trialkoxysilane and trimethylsilane derivatives – the latter is used in this work.

However, these methodologies present some challenges which limit their wider application. For instance, the high reactivity of Grignard reagents is not well tolerated by many functional groups, limiting their utility to labelling functionally simple substrates.^3^ In addition, Grignard reagents are very sensitive to moisture or reaction with atmospheric CO_2_, even if great care is used in the storage and use of these reagents, leading to isotopic dilution of [^11^C]CO_2_ and concomitant low molar activity (*A*_m_) of ^11^C-labelled products.

Compared to Grignard reagents, boronic esters have greater stability to atmospheric CO_2_ and moisture which broadens their use for radiolabelling aromatic and heteroaromatic compounds.^4^ However, the radiolabelling of the latter class of compounds (*e.g.* pyridyl, pyrazyl and thienyl boronic ester derivatives) is inconsistent and gives low-moderate radiochemical yields (RCYs: 3–69%).4*a* Recently, a dynamic carbon isotope exchange (isotopic enrichment) of carboxylates using [^13^C]CO_2_ and [^14^C]CO_2_ has been reported; however, the applicability of this methodology to carbon-11 chemistry would require an extensive study on the range of molar activity that could be obtained.^5^

Based on a search of the traditional synthetic chemistry literature, improved methods for the ^11^C-carboxylation of aryl and heteroaryl groups might be achieved by the use of trialkoxysilyl and trimethylsilyl derivatives *via* a so-called copper-catalysed desilylative carboxylation reaction.^6^ Arylsilanes reacted readily with a fluoride anion source, such as cesium fluoride (CsF), potassium fluoride (KF), and tetramethylammonium fluoride (Me_4_NF), to form a pentavalent silicate.^6^,^7^ The pentavalent silicate was then converted in the presence of a copper catalyst to an aryl copper intermediate which reacted with non-radioactive CO_2_ in moderate to excellent yields (27–99%).^6^,^7^ Varying the substitution patterns of the aromatic ring with electron-withdrawing or electron donating groups did not alter the efficiency of substrate carboxylation.6a–c Excellent results were also reported for the carboxylation of heteroaromatic compounds, such as thiophenyl, pyridyl and furanyl silane derivatives, and their derivatization to ester products (89–93%).6b,c

Compared to the traditional ^11^C-carboxylation methodologies, the use of silyl derivatives would provide greater air and moisture stability and therefore easier handling and storage. Moreover, trimethylsilyl and trialkoxysilyl precursors are readily obtained *via* a plethora of synthetic reagents: Grignard or organolithium reagents^8^ or functionalization of arylamides,^9^ aryl acyl fluorides,^10^ aryl esters,^11^ and aryl cyanides^12^*via* transition metals (nickel, copper, and ruthenium).

With the aim of developing more robust and versatile ^11^C-carboxylation methodologies, we herein present the development of a novel ^11^C-carboxylation protocol involving the use of arylsilyl derivatives. The carbon-11 labelled carboxylic acids were obtained in short synthesis times, with high molar activities and with broad applicability to a range of trimethylsilane and trialkoxysilane derivatives.

2-(Thienyl)trimethylsilane (**1a**, Scheme 2) was initially chosen as a model substrate for cyclotron-produced [^11^C]CO_2_ carboxylation reactions. Liu *et al.* reported that the combination of CsF and 18-crown-6 in the presence of CO_2_ (1 atm) allowed the carboxylation of trimethylsilane derivatives in high yields.^13^

**Scheme 2:**
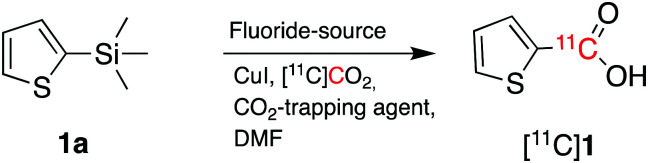
Radiosynthetic approach to radiolabelled carbon-11 labelled carboxylic acids from cyclotron-produced [^11^C]CO_2_.

As a starting point, we applied the same approach of using CsF and 18-crown-6 (CsF–crown) in the presence of [^11^C]CO_2_ to carboxylate **1a**. However, when **1a** (100 μmol, 1 equiv.) was reacted with [^11^C]CO_2_ for 5 minutes at 100 °C in dimethylformamide (DMF), no [^11^C]**1** was formed and the resulting [^11^C]CO_2_ trapping efficiency (TE, see footnote ‡
‡Radiochemical yield (RCY) of the crude product has been determined by analytical radio-HPLC (non-isolated). The trapping efficiency (TE) has been calculated as a ratio of the decay corrected radioactivity in the vial and the total radioactivity produced by the cyclotron.) was poor (entry 1, Table 1).

**Table 1:** Reaction conditions and optimisation for the synthesis of [^11^C]**1** using DBU as a trapping agent (see footnote ‡)

Entry^*a*^	Fluoride source (eq.)	Additive (eq.)	DBU (eq.)	CuI (%)	TE (%)	RCY [^11^C]**1** (%)
1^*b*^	CsF (3)	18-Crown-6 (3)	—	—	6	0
2^*b*^	CsF (3)	18-Crown-6 (3)	—	10	99	0
3^*b*^	CsF (3)	18-Crown-6 (3)	0.6	10	77	0
4^*b*^	KF (3)	K2.2.2 (3)	0.6	10	96	21
5	KF (0.5)	K2.2.2 (0.5)	0.6	10	63 ± 14	41 ± 9
6	KF (0.25)	K2.2.2 (0.25)	0.6	10	52 ± 5	53 ± 23
7^*a*^ ^,^^*c*^	KF (0.5)	K2.2.2 (0.5)	0.6	10	77	40
8^*c*^	KF (0.25)	K2.2.2 (0.25)	0.6	10	67 ± 13	55 ± 7
9^*b*^ ^,^^*d*^	KF (0.25)	K2.2.2 (0.25)	0.6	10	37	0
10	KF (0.25)	K2.2.2 (0.25)	0.9	10	86 ± 5	28 ± 11
11	KF (0.25)	K2.2.2 (0.25)	0.6	20	61 ± 31	44 ± 26
12^*b*^ ^,^^*c*^ ^,^^*e*^	KF (0.25)	K2.2.2 (0.25)	0.6	10	7	15

^*a*^ *n* = 3.

^*b*^ *n* = 1.

^*c*^140 °C.

^*d*^70 °C.

^*e*^THF.

This might be due to the poor reactivity of the pentavalent silicate intermediate and/or the absence of any [^11^C]CO_2_ trapping agent. The transmetallation of hypervalent silicates with copper catalysts (10%), however, has been shown to form aryl copper intermediates that readily react with non-radioactive CO_2_.6*a* Despite this finding, in our hands, the addition of 10% CuI to the reaction mixture did not promote the formation of [^11^C]**1** (entry 2, Table 1). Moreover, the addition of a [^11^C]CO_2_ trapping agent (1,8-diazabicyclo[5.4.0]undec-7-ene, DBU, 0.6 equiv.) did not favour the formation of [^11^C]**1** either, although the TE increased from 6% to 77% (entry 1 *versus* 3).

We subsequently focused on selecting alternative fluoride sources as CsF is highly hygroscopic and poorly soluble in organic solvents – even in the presence of 18-crown-6, which might have hampered the formation of [^11^C]**1**. KF was investigated as a fluoride source as it has previously been used for the carboxylation of aryltrimethylsilanes; however, due to the low reactivity of KF in organic solvents the corresponding carboxylic acid derivative was only obtained with a low to moderate yield (17–74%).6c,^14^ To increase the reactivity of KF in organic solvents, we opted to explore the use of the polyether kryptofix (K2.2.2) to form a K^+^–cryptand complex.

Interestingly, replacing CsF–crown with KF–K2.2.2 improved the formation of [^11^C]**1** (100 °C, 5 minutes) giving radiochemical yields (RCY, see footnote ‡) of 21% and high TE (96%, entry 4).^15^

In order to further increase the RCY of [^11^C]**1**, an optimization process was subsequently performed by modifying: (i) the amount of fluoride source, (ii) the reaction temperature, (iii) the amount of trapping reagent, (iv) the amount of copper catalyst and (v) the solvent.

The effect of the equivalents of fluoride source was initially investigated. Lowering the equivalents of the KF–K2.2.2 complex from 3 to 0.5 and 0.25 equivalents, and keeping the temperature at 100 °C, enhanced the RCY of [^11^C]**1** (21% with 3 equiv., 41% with 0.5 equiv., and 53% with 0.25 equiv., entries 4–6). A similar trend was obtained at 140 °C (40% with 0.5 equiv. and 55% with 0.25 equiv., entries 7 and 8).

Additionally, we observed that higher temperatures favoured the formation of [^11^C]**1** – either when 0.5 equivalents (41% at 100 °C *versus* 40% at 140 °C, entries 5 and 7) or 0.25 equivalents (53% at 100 °C *versus* 55% at 140 °C, entries 6 and 8) of the KF–K2.2.2 complex were used. Conversely, when lowering the temperature to 70 °C, [^11^C]**1** was not obtained (entry 9) as high temperature is needed for the activation of the desilylation reaction. Similarly, the carboxylation of arylsilane derivatives with CO_2_ is promoted by high temperature.6*a*

Increasing the amount of the trapping agent (DBU) from 0.6 to 0.9 equivalents halved the RCY of [^11^C]**1** (53% *versus* 28%, entries 6 and 10, respectively). Similarly, increasing the content of CuI from 10% to 20% did not markedly affect the RCY of [^11^C]**1** (53% at 10% *versus* 44% at 20%, entries 6 and 11).

The use of a different solvent was investigated. Using tetrahydrofuran (THF) instead of DMF had a negative effect on reactivity, with the RCY of [^11^C]**1** dropping to 15% (entry 12).

Optimal conditions were obtained when **1a** (100 μmol, 1 equiv.) was reacted with the cyclotron-produced [^11^C]CO_2_ at 140 °C in the presence of 0.25 equiv. of KF–K2.2.2, 10% of CuI and DMF (entry 8, Table 1).

Aiming to further increase the RCY of [^11^C]**1**, DBU was substituted with BEMP as a CO_2_ trapping agent. Although no significant difference in RCY was observed at 100 °C (33 ± 15% with BEMP, entry 1, Table 2 *versus* 53 ± 23% with DBU, entry 6, Table 1), higher yields of [^11^C]**1** were obtained when the temperature was increased to 140 °C (93 ± 6% with BEMP, entry 2, Table 2 *versus* 55 ± 7% with DBU, entry 8, Table 1). Using BEMP over DBU, a significant increase in TE was observed at 100 °C (84% with BEMP, entry 2, Table 2 *versus* 52% with DBU, entry 6, Table 1) and at 140 °C (89% with BEMP, entry 2, Table 2 *versus* 67% with DBU, entry 8, Table 1).

**Table 2:** Reaction conditions and optimisation for the synthesis of [^11^C]**1** using BEMP as a trapping agent (see footnote ‡)

Entry^*a*^	KF (eq.)	K2.2.2 (eq.)	BEMP (eq.)	CuI (%)	TE (%)	RCY [^11^C]**1** (%)
1^*b*^	0.25	0.25	0.6	10	84 ± 3	33 ± 15
2	0.25	0.25	0.6	10	89 ± 8	93 ± 6
3^*c*^	0.25	0.25	0.6	10	76 ± 12	58 ± 9
4	0.25	0.25	—	10	6 ± 2	95 ± 1
5	0.25	0.25	0.6	—	76 ± 22	24 ± 18
6^*d*^	—	0.25	0.6	10	40, 30	0
7^*e*^	0.25	—	0.6	10	48	0
8^*f*^	0.125	0.125	0.6	10	55 ± 15	47 ± 9
9^*g*^	0.25	0.25	0.6	10	12 ± 5	20 ± 7
10^*e*^ ^,^^*h*^	0.25	0.25	0.6	10	50	0

^*a*^ *n* = 3.

^*b*^100 °C.

^*c*^2.5 minutes.

^*d*^ *n* = 2.

^*e*^ *n* = 1.

^*f*^ **1a** (50 μmol).

^*g*^THF.

^*h*^MeCN.

Encouraged by these results, BEMP was used as a trapping agent for the following experiments which initially focused on the effect of shorter reaction times. Halving the reaction time from 5 to 2.5 minutes resulted in reducing the RCY of [^11^C]**1** (58% at 2.5 min *versus* 93% at 5 min, entries 2 and 3, Table 2).

To understand the role of each reagent in the reaction mechanism, experiments were conducted with the omission of key reagents (KF, K2.2.2, BEMP, or CuI) from the reaction mixture. Removing BEMP yielded [^11^C]**1** with high RCY but with a significantly lower TE (6% without BEMP and 89% with BEMP, entries 2 and 4). Removing CuI yielded [^11^C]**1** with significantly lower RCY (93% with CuI and 24% without CuI, entries 2 and 5) but the TE was not clearly affected. Notably, [^11^C]**1** was not formed at all when KF or K2.2.2 was eliminated from the reaction mixture (entries 6 and 7, respectively). Similarly, when the amount of **1a** and KF–K2.2.2 was halved, the RCY of [^11^C]**1** was reduced 2-fold (47%, entry 8). These results highlight the primary role of the concentration of **1a** and fluoride source to promote the formation of a highly nucleophilic intermediate, which is stabilized by the copper catalyst. The effect of the solvent was also investigated during the optimisation of the reaction conditions. The use of THF and acetonitrile (MeCN) gave low or zero RCYs of [^11^C]**1** (20% in THF and 0% in MeCN, entries 9 and 10, Table 2).

The results presented in Tables 1 and 2 show that the RCY of [^11^C]**1** is maximized when 100 μmol of **1a** is reacted with 0.6 equiv. of BEMP, 0.25 equiv. of KF–K2.2.2 and 0.1 equiv. of CuI in DMF for 5 minutes at 140 °C (entry 2, Table 2). Following this protocol, for [^11^C]**1** the isolated (by semipreparative HPLC) decay-corrected-RCY of 17 ± 5% and an *A*_m_ of 3.1 ± 0.4 Gbq μmol^–1^ at the end of bombardment (EOB) were obtained starting from 2.30 ± 0.3 GBq of [^11^C]CO_2_.§
§This work describes a method development study using short, low current, cyclotron irradiations where obtaining high molar activities (*A*_m_) was not the main focus. Assuming that the stable ^12^C carrier content would be in the same range for a standard clinical [^11^C]CO_2_ production (30 GBq), it is estimated that a molar activity of ∼45 GBq μmol^–1^ would be obtained.


The reaction conditions were subsequently kept constant while studying the substrate scope of additional trialkoxysilyl and trimethylsilyl compounds. Initially, the effect of silyl substituents other than the trimethyl silyl moiety on the thienyl ring was explored using a triethoxysilyl substituent (triethoxy-2-thienylsilane, **1b**, Table 3). Both precursors **1a** and **1b** yielded the corresponding [^11^C]**1** with high RCY (93% and 90%, respectively). However, the use of **1b** resulted in lower TE (57%, entry 1, Table 3) compared with **1a** (89%, entry 2, Table 2).

**Table 3:** Radiolabelling aromatic ^11^C-carboxylic acids ([^11^C]**1–6**) with [^11^C]CO_2_ and silyl derivatives (see footnote ‡)

Entry^*a*^	Reagent		R	Product	Temp. (°C)	TE (%)	RCY (%)
1	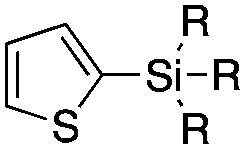	**1b**	OEt	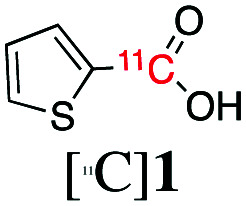	140	57 ± 18	90 ± 4
2	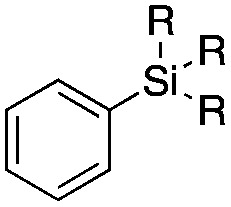	**2a**	Me	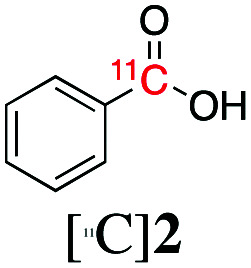	140	13 ± 8	0
3		**2b**	OEt		140	76 ± 8	84 ± 2
4	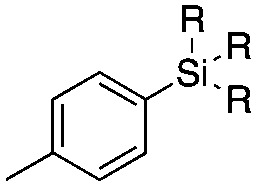	**3a**	Me	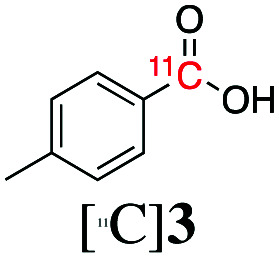	140	15 ± 8	0
5		**3b**	OEt		140	81 ± 2	78 ± 2
6	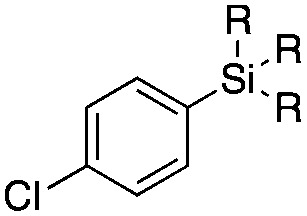	**4a**	Me	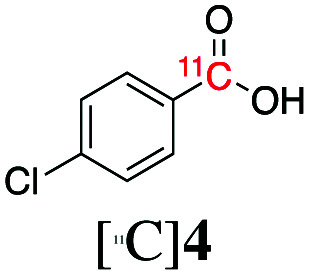	140	23 ± 15	18 ± 7
7	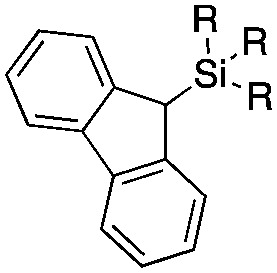	**5a**	Me	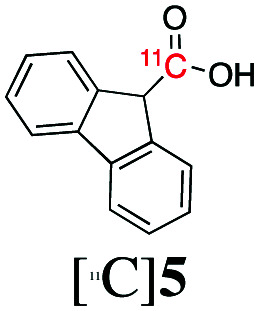	140	40 ± 1	87 ± 6
8	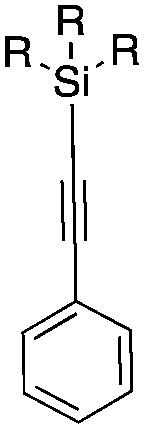	**6a**	Me	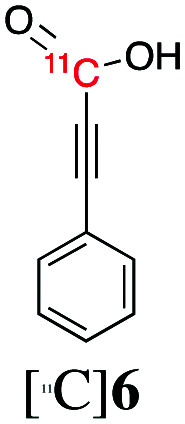	140	5 ± 4	0
9					100	3 ± 2	9 ± 8
10					30	21 ± 12	19 ± 15

^*a*^ *n* = 3.

Next, we directed our attention on radiolabelling other ^11^C-labelled aromatic carboxylic acids such as [^11^C]benzoic acid ([^11^C]**2**, entries 2 and 3) and [^11^C]*p*-toluic acid ([^11^C]**3**, entries 4 and 5) using trimethyl silyl (**2a** and **3a**) and the triethoxysilyl (**2b** and **3b**) precursors. In contrast to that observed with [^11^C]**1**, the trimethyl silyl derivatives showed a different reactivity to triethoxysilyl analogues. **2b** and **3b** produced the corresponding carbon-11 labelled carboxylic acids with high RCYs (RCY of [^11^C]**2** = 84%, entry 3; RCY of [^11^C]**3** = 78%, entry 5), whereas the trimethylsilyl derivatives, **2a** and **3a**, did not form the desired products (entries 2 and 4). As expected, the low reactivity of benzyl-trimethylsilyl substrates was also observed using 1-chloro-4-(trimethylsilyl)benzene (**4a**), yielding only small amounts of [^11^C]**4** (18%, entry 6). Further studies focused on non-aromatic silane precursors such as fluorene and alkyne derivatives (entries 7–10). The radiolabelling of a fluorene moiety (**5a**) was effective, producing [^11^C]fluorene-9-carboxylic acid ([^11^C]**5**) with high RCY (87%, entry 7). The radiolabelling of prop-1-yn-1-ylbenzene (**6a**) to [^11^C]3-phenylpropiolic acid ([^11^C]**6**), instead, was ineffective at 140 °C (entry 8) and 100 °C (entry 9). However, lowering the temperature to 30 °C yielded [^11^C]**6**, although with low RCY (19%, entry 10).

To demonstrate that the arylcopper intermediates were obtained by the KF–K2.2.2-mediated desilylation of trimethylsilyl derivatives, we replaced [^11^C]CO_2_ with [^11^C]CH_3_I. [^11^C]**7** was obtained by direct aromatic ^11^C-methylation of **1a** (Scheme 3), with a RCY of 16 ± 4% (*n* = 3). Although this method has not been optimised here, we note a potential application of this strategy as an alternative route to produce ^11^C-methylaromatic radiopharmaceuticals such as (15R)-[^11^C]TIC, [^11^C]MNQP, [^11^C]M-MTEB, [^11^C]celecoxib, [^11^C]cibbi-772, and [^11^C]UCB-J by direct aromatic ^11^C-methylation.^2^

**Scheme 3:**
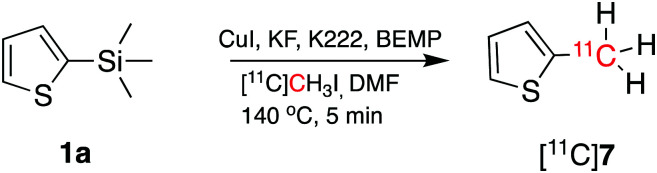
Aromatic ^11^C-methylation of **1a** using [^11^C]CH_3_I to obtain [^11^C]**7**.

In summary, we have developed a novel carbon-11 reaction using cyclotron-produced [^11^C]CO_2_ and aryltrimethylsilanes and aryltrialkoxysilanes to obtain ^11^C-carboxylic acid derivatives. Aryltrimethylsilanes and aryltrialkoxysilanes are activated by a fluoride source (KF–K2.2.2) and a copper catalyst which readily react with cyclotron-produced [^11^C]CO_2_. We have also expanded the use of activated aryltrimethylsilanes as nucleophilic compounds for aromatic ^11^C-methylation using [^11^C]CH_3_I. This one-pot methodology, similar to other one-pot reactions using ^11^C-syntons,^16^ has the compatibility to be fully automated using a commercial radiochemistry synthesis module. The application of silane-mediated ^11^C-carboxylation reactions has the potential to be an alternative route to produce a plethora of radiopharmaceuticals bearing an aryl carboxylic acid such as [^11^C]bexarotene, [^11^C]eprosartan, and [^11^C]Am80, or an aryl carboxylic acid that is subsequently converted into an ^11^C-amide by an amide-coupling reaction such as [^11^C]raclopride, [^11^C]olaparib, [^11^C]JNJ-31020028, [^11^C]FIMX, [^11^C]tubastatin A, and [^11^C]AZ11136118.^2^

This work was supported by Medical Research Council (MRC, MR/K022733/1) and European Commission, FP7-PEOPLE-2012-ITN (316882, RADIOMI). The authors acknowledge financial support from the Department of Health *via* the National Institute for Health Research (NIHR) Biomedical Research Centre at Guy's & St Thomas’ NHS Foundation Trust and King's College London and the Centre of Excellence in Medical Engineering funded by the Wellcome Trust and EPSRC under grant number WT 203148/Z/16/Z. Igor Camargo Fontana acknowledges financial support from the Coordenação de Aperfeiçoamento de Pessoal de Nível Superior (CAPES, project number: 88887.185806/2018-00).

## Conflicts of interest

There are no conflicts to declare.

## Supplementary Material

Supplementary information
