# Supplementary material for: Carbon-11 carboxylation of trialkoxysilane and trimethylsilane derivatives using [11C]CO2
Source: Chem Commun (Camb). 2020 Mar 25;56(34):4668–71. doi: 10.1039/d0cc00449a (PMC7384297; doi:10.1039/d0cc00449a)
Supplement: Supplementary file 1 [file CC-056-D0CC00449A-s001.pdf]

Electronic Supplementary Material (ESI) for ChemComm.
This journal is © The Royal Society of Chemistry 2020

Carbon-11 carboxylation of trialkoxysilane and trimethylsilane derivatives using [^{11}C]CO_2

Salvatore Bongarzone,[a] Nicola Raucci,[a] Igor Camargo Fontana,[a] Federico Luzi[a] and Antony D. Gee[a]

[a]School of Biomedical Engineering & Imaging Sciences, King's College London, King's Health Partners, St Thomas' Hospital, London SE1 7EH, United Kingdom

Supplementary Information

Table of Contents

General Method and Materials 2

Carbon-11 Radiochemistry 2

[^{11}C]CO_2 Production 2

Description of the system 2

Description of the carbon-11 carboxylation 3

Molar Activity calculation of [^{11}C]1 3

Description of the carbon-11 methylation to obtain [^{11}C]7 3

Quality control of compounds [^{11}C]1-[^{11}C]7 4

Semipreparative HPLC method for the purification of [^{11}C]1. 5

Fig. S1 5

Fig. S2. 5

Fig. S3. 6

General Method and Materials

2-Thiophencarboxylic acid (**1,** 99%), benzoic acid (**2**, 99%), toluic acid (**3,** 99%), fluorene-9-carboxylic acid (**4**, 96%), phenylpropiolic acid (**5**, 99%), 4-chlorobenzoic acid (**6**, 99%), 2-methyltiophene (**7**, 99%), trimethyl-2-thienylsilane (**1a**, 97%), triethoxy-2-thienylsilane (**1b**, 97%), trimethyl(phenyl)silane (**2a**, 99%), triethoxy(phenyl)silane (**2b**, 98%), trimethyl-p-tolylsilane (**3a**, 97%), triethoxy-p-tolylsilane (**3b**, 97%), 9-trimethylsilylfluorene (**4a**, 98%), 1-phenyl-2-trimethylsilylacetilene (**5a**, 99%), 1-chloro-4(trimethylsilyl)benzene (**6a**, 98%), potassium fluoride (KF, 99%), 4,7,13,16,21,24-hexaoxa-1,10-diazabicyclo[8.8.8]hexacosane (K2.2.2, 99%), 2-*tert*-butylimino-2-diethylamino-1,3-dimethylperhydro-1,3,2-diazaphosphorine (BEMP, 98%), cesium fluoride (CsF, 99%), 1,4,7,10,13,16-hexaoxacyclooctadecane (18-crown-6, 99%), copper(I) iodide (99%), *N*,*N*-dimethylformamide (DMF, 99%), 1,8-diazabicyclo[5.4.0]undec-7-ene (DBU, 98%), tetrahydrofuran (THF, 99%), trifluoroacetic acid (TFA, 99%), acetonitrile (MeCN, for HPLC ≥99%). All chemicals and dry solvents were purchased from Sigma-Aldrich, Alfa Aesar, Merck, Fisher Scientific and Acros Organics. TE, RCP, RCY and molar activity values are reported as mean ± standard deviation.

Carbon-11 Radiochemistry

Preparation of the Vial

An oven-dried vial (KX Microwave Vials, 5 mL) and a crimp cap (Fisherbrand, centre hole with 3.0 mm PTFE seal aluminium silver 20 mm, part # 10132712) were used. The vials were prepared in a glovebox (Plas-Labs, Inc. 815 PGB Series) under nitrogen atmosphere and controlled CO_2 levels (lower than 30 ppm).

$[^{11}C]CO_2$ Production

$[^{11}C]CO_2$ was produced using a Siemens RD112 cyclotron by the 11 MeV proton bombardment of nitrogen (+0.5% O_2) gas via the $^{14}N(p,\alpha)^{11}C$ reaction. The cyclotron-produced $[^{11}C]CO_2$ was bubbled in a stream of helium gas with a flow rate of 60 mL/min post target depressurisation directly into a reaction v-vial (time from end of bombardment (EOB) to end of delivery (EOD) = 1 minute and 50 seconds).

Description of the system

The set up was implemented on an Eckert & Ziegler system (Modular-Lab Standard) and included two switching valves and a heating block. All gas transfer lines were fabricated from PTFE tubing (length: 10–30 cm, O.D.: 0.79 x 0.4 in., I.D.: 1/32 x 0.16 in.). A P_2O_5 trap and one-way valve (BRAUN, normally closed backcheck valve, part # 415062) were placed before the vial. The outlet gas line of the vial was connected to a cartridge (Biosys Solutions Ltd, Fritted Empty MiniSpeed Cartridges, part # 2447) filled with ascarite® (Sigma-Aldrich, 1310-73-2) to trap unreacted $[^{11}C]CO_2$. A tedlar® gas waste bag was placed at the outlet of the ascarite's cartridge to prevent any gaseous emission.

Description of the carbon-11 carboxylation

A cyclotron beam current of 5 µA was maintained for a bombardment time of 1 minute for all reaction optimization experiments producing ~ 300 MBq of carbon-11 at EOD.

[^{11}C]CO_2 (carried by helium gas) was bubbled directly from the target into a reaction vial containing aryltrimethylsilane or aryltrialkoxysilanes and reagents described in **Tables 1-3** at 0 °C. The outlet gas line of the vial was connected to an Ascarite® cartridge. After the delivery of [^{11}C]CO_2 (1.75 minutes from end of bombardment) the temperature was increased to 30, 70, 100, 140 °C for 2.5-5 minutes. At five minutes, the system was flushed with helium (60 ml/min) for 20 seconds. Thereafter, the reaction was cooled at 0 °C and quenched with a solution of 0.5% trifluoroacetic acid TFA (CF_3COOH) in MeCN/H_2O (1:1, 1 mL). The amount of radioactivity in the Ascarite® and vial were measured (to determine the trapping efficiency, TE), and an aliquot of the crude mixture analysed by radio-HPLC to determine the radiochemical purity, RCP.

Molar Activity calculation of [^{11}C]1

Eleven samples of **1** at different concentrations (1.15-0.011 µmol/mL) were analysed by HPLC to obtain a calibration curve of the peak area (mAU*s) versus µmol/mL. The peak areas of **1** were averaged and plotted in function of the corresponding µmol/mL (**Figure S2**).

[^{11}C]**1** was produced following the procedure of entry 2 (**Table 2)** by starting from 2.30 ± 0.3 GBq of [^{11}C]CO_2. After quenching the reaction, [^{11}C]**1** was purified by semipreparative HPLC and the peak corresponding to [^{11}C]**1** collected.

The radioactivity in 1.00 mL of solution containing the purified [^{11}C]**1** was determined. An aliquot of purified [^{11}C]**1** (20 µL) was analysed by analytical radio-HPLC (**Figure S3**) and the UV peak corresponding to **1** was integrated. The area of the UV peak was used to determine the µmol/mL of the associated ^{12}C-carrier content for [^{11}C]**1** from the equation of the calibration curve. The molar activity (A_m) of [^{11}C]**1** was calculated to be 3.1 ± 0.4 GBq/µmol at EOD (n = 3).

Description of the carbon-11 methylation to obtain [^{11}C]7

The [^{11}C]CO_2 was transferred in a stream of helium at 70 mL/min to a GE TRCAERLab® FX MeI module ($t_{delivery}$ = 1.75 minutes). [^{11}C]CH_3I was produced by gas phase conversion from [^{11}C]CO_2 and transferred in a vial containing 1 mL of DMSO.

A DMSO solution of [^{11}C]CH_3I (2-4 MBq) was transferred into a reaction vial containing **1a** (0.1 mmol, 1 equiv.), KF (0.25 equiv.), K2.2.2, (0.25 equiv.), BEMP (0.6 equiv.) and CuI (10%) in 500 µL of DMF at 0 °C. The temperature was then increased to 140 °C for 5 minutes. Thereafter, the reaction was cooled at 0 °C and quenched with a solution of 0.5% TFA in MeCN/H_2O 1:1. An aliquot of the crude mixture analysed by radio-HPLC to determine the radiochemical purity (RCP).

Quality control of compounds [^{11}C]1-[^{11}C]7

HPLC analysis was performed on an Agilent 1200 system equipped with a UV detector (λ=254 nm) and a β+-flow detector coupled in series. A reverse-phase column (Phenomenex Luna-C18, 4.6 x 150 mm, 5 µm) was used with a flow rate of 1 mL/min.

Identification of all radioactive products was confirmed by co-elution of ([^{11}C]**1**-[^{11}C]**7**) with the corresponding non-radioactive compounds (**1-7**).

Compounds [^{11}C]**1** and [^{11}C]**7**

The HPLC method was isocratic between 0-5.5 minutes (CH_3CN + 0.5% TFA: H_2O + 0.5% TFA, 25:75), gradient between 5.5-6 minutes (25:75 to 0:100), isocratic between 6-9 minutes (0:100), gradient between 9-10 minutes (0:100 to 25:75), isocratic between 10-13 minutes (25:75).

[^{11}C]**1** t_R= 5 minutes and 20 seconds (**Figure S1**).

[^{11}C]**7** t_R= 8 minutes and 40 seconds.

Compound [^{11}C]**2**

The HPLC method was isocratic between 0-9 minutes (CH_3CN + 0.5% TFA: H_2O + 0.5% TFA, 25:75), gradient between 9-10 minutes (25:75 to 0:100), isocratic between 10-13 minutes (0:100), gradient between 13-14 minutes (0:100 to 25:75) and isocratic between 14-17 minutes (25:75).

t_R= 7 minutes and 30 seconds.

Compounds [^{11}C]**3** and [^{11}C]**5**

The HPLC method was isocratic between 0-5.5 minutes (CH_3CN + 0.5% TFA: H_2O + 0.5% TFA, 35:65), gradient between 5.5-9 minutes (35:65 to 0:100), isocratic between 9-12 minutes (0:100), gradient between 12-13 minutes (0:100 to 35:65), isocratic between 13-16 minutes (35:65).

[^{11}C]**3**: t_R= 6 minutes and 20 seconds.

[^{11}C]**5**: t_R= 7 minutes and 7 seconds.

Compound [^{11}C]**4**

The HPLC method was isocratic between 0-5.5 minutes (CH_3CN + 0.5% TFA: H_2O + 0.5% TFA, 50:50), gradient between 5.5-9 minutes (50:50 to 0:100), isocratic between 9-12 minutes (0:100), gradient between 12-13 minutes (0:100 to 50:50), isocratic between 13-16 minutes (50:50).

t_R= 5 minutes and 46 seconds.

Compound [^{11}C]**6**

The HPLC method was isocratic between 0-5.5 minutes (CH_3CN + 0.5% TFA: H_2O + 0.5% TFA, 45:55), gradient between 5.5-9 minutes (45:55 to 0:100), isocratic between 9-12 minutes isocratic (0:100), gradient between 12-13 minutes (0:100 to 45:55), isocratic between 13-16 minutes (45:55).

t_R= 5 minutes and 30 seconds.

Semipreparative HPLC method for the purification of [^{11}C]1.

HPLC analysis was performed on an Agilent 1200 system equipped with a UV detector (λ=254 nm) and a β+-flow detector coupled in series. A reverse-phase column (Phenomenex Luna-C18, 10 x 250 mm, 5 µm) was used with a flow rate of 4 mL/min.

The HPLC method was isocratic between 0 – 10.4 minutes (CH_3CN + 0.5% TFA: H_2O + 0.5% TFA, 25:75), gradient between 10.4-11.8 minutes (25:75 to 0:100), isocratic between 11.8-17.7 minutes (0:100), gradient between 17.7-19.69 minutes (0:100 to 25:75), isocratic between 19.69-26 minutes (25:75). t_R= 11 minutes and 48 seconds.

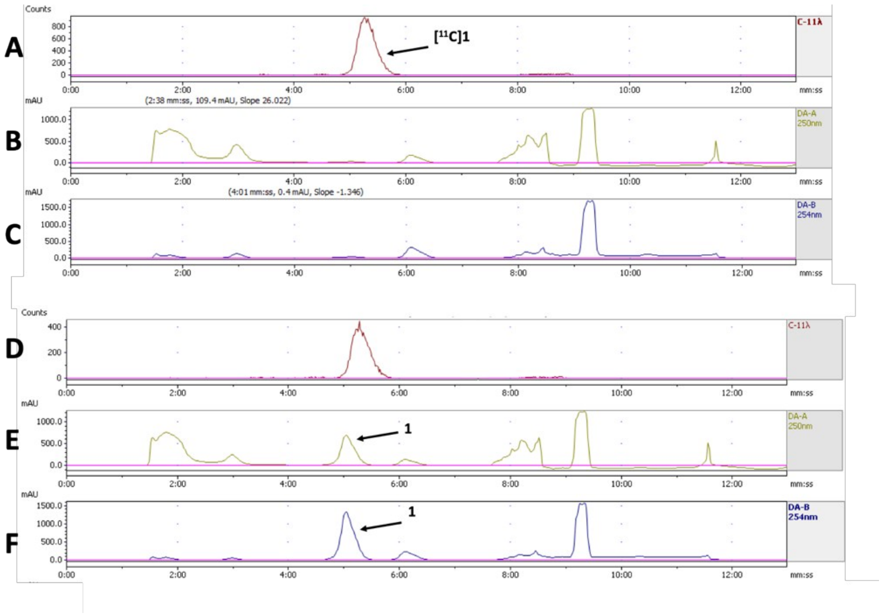

Fig. S1 A) Radio-HPLC chromatogram of crude [^{11}C]**1** (experiment Table 1 entry 2). **B** and **C)** UV chromatograms of crude [^{11}C]**1** at 250 and 254 nm, respectively. **D)** Radio-HPLC chromatogram of crude [^{11}C]**1** co-injected with **1**. **E** and **F)** UV chromatograms of crude [^{11}C]**1** co-injected with **1** at 250 and 254 nm, respectively. The difference between UV peaks (retention time (t_R) = 5 minutes and 7 seconds) and radioactivity peaks (t_R = 5 minutes and 20 seconds) is 13 seconds, consistent with the expected delay time between detectors (13 seconds).

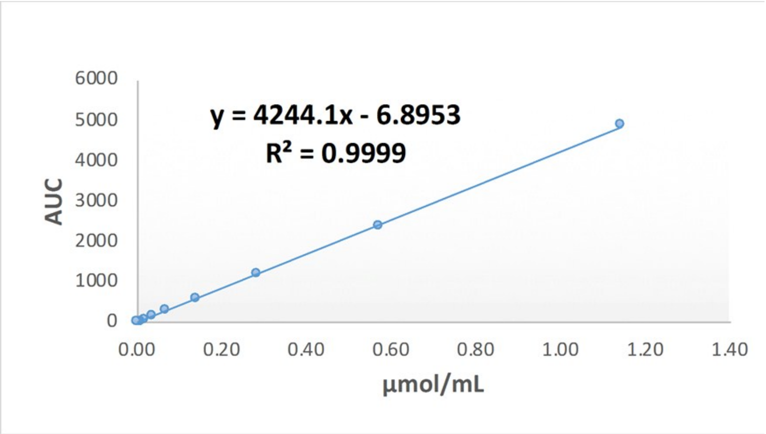

Fig. S2 Calibration Curve for **1**.

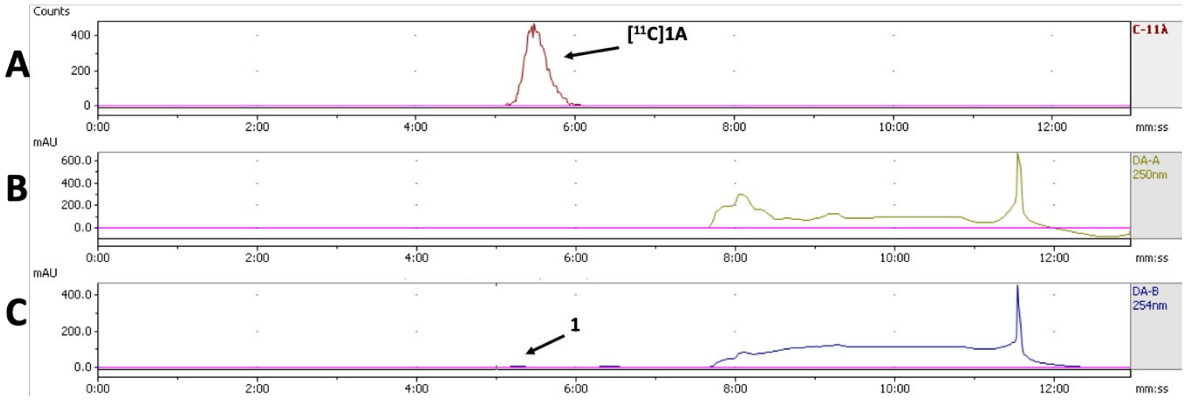

Fig. S3 A) Radio-HPLC chromatogram of purified [^{11}C]**1**. [^{11}C]**1** has been purified by semipreparative HPLC column. **B** and **C)** UV chromatograms of HPLC-purified [^{11}C]**1** at 250 and 254 nm, respectively. The difference between UV peaks (retention time (t_R) = 5 minutes and 15 seconds) and radioactivity peaks (t_R = 5 minutes and 28 seconds) is 13 seconds consistent with the expected delay time between detectors (13 seconds).
